# Proteome Response of *Tribolium castaneum* Larvae to *Bacillus thuringiensis* Toxin Producing Strains

**DOI:** 10.1371/journal.pone.0055330

**Published:** 2013-01-25

**Authors:** Estefanía Contreras, Carolina Rausell, M. Dolores Real

**Affiliations:** Departamento de Genética, Facultad de Ciencias Biológicas, Universidad de Valencia, Burjassot, Valencia, Spain; University of Tennessee, United States of America

## Abstract

Susceptibility of *Tribolium castaneum* (Tc) larvae was determined against spore-crystal mixtures of five coleopteran specific and one lepidopteran specific *Bacillus thuringiensis* Cry toxin producing strains and those containing the structurally unrelated Cry3Ba and Cry23Aa/Cry37Aa proteins were found toxic (LC_50_ values 13.53 and 6.30 µg spore-crystal mixture/µL flour disc, respectively). Using iTRAQ combined with LC-MS/MS allowed the discovery of seven novel differentially expressed proteins in early response of Tc larvae to the two active spore-crystal mixtures. Proteins showing a statistically significant change in treated larvae compared to non-intoxicated larvae fell into two major categories; up-regulated proteins were involved in host defense (odorant binding protein C12, apolipophorin-III and chemosensory protein 18) and down-regulated proteins were linked to metabolic pathways affecting larval metabolism and development (pyruvate dehydrogenase Eα subunit, cuticular protein, ribosomal protein L13a and apolipoprotein LI-II). Among increased proteins, Odorant binding protein C12 showed the highest change, 4-fold increase in both toxin treatments. The protein displayed amino acid sequence and structural homology to *Tenebrio molitor* 12 kDa hemolymph protein b precursor, a non-olfactory odorant binding protein. Analysis of mRNA expression and mortality assays in Odorant binding protein C12 silenced larvae were consistent with a general immune defense function of non-olfactory odorant binding proteins. Regarding down-regulated proteins, at the transcriptional level, pyruvate dehydrogenase and cuticular genes were decreased in Tc larvae exposed to the Cry3Ba producing strain compared to the Cry23Aa/Cry37Aa producing strain, which may contribute to the developmental arrest that we observed with larvae fed the Cry3Ba producing strain. Results demonstrated a distinct host transcriptional regulation depending upon the Cry toxin treatment. Knowledge on how insects respond to Bt intoxication will allow designing more effective management strategies for pest control.

## Introduction

The entomophatogenic bacterium *Bacillus thuringiensis* (Bt) represents an environmentally safe alternative for pest control producing parasporal inclusions, which contain one or several insecticidal proteins. The greatest variety of toxins found in the crystals of Bt are proteins of the Cry (for Crystal) or Cyt (for Cytotoxic) type [1]. The largest group of Cry toxins corresponds to the 3-domain Cry proteins (Cry-3D), including at least 40 different groups with more than 200 different gene sequences [2]. Other Cry proteins display no homology to the Cry-3D proteins, such as Cry35Ab and Cry36Aa proteins, Cry34Ab and Cry35Ab proteins, and the coleopteran active Cry23Aa and Cry37Aa proteins, homologous to BinA and BinB binary toxins or Mtx toxins from *Bacillus sphaericus* [3].

The red flour beetle, *Tribolium castaneum* (Tc), is a major global pest of stored grain, cereal products, and peanuts for human consumption [4]. This coleopteran insect, readily adaptable to all classes of insecticides, is an ideal subject for the identification of new pesticide targets for which many genetic and genomics tools have been developed and it has become the genetic model for agriculturally important coleopteran species [5]. Tc bioassays with Bt toxins, Cry3Aa, Cry8Ea, Cry8Fa, Cry8Ga, Cry23Aa/Cry37Aa, Cyt2Ca, Cry34Ab/Cry35Ab and Cry1F, have been carried out showing that Cry23Aa/Cry37Aa and Cyt2Ca were active, Cry3Aa intermediate-active and Cry8Ea, Cry8Fa, Cry8Ga, Cry34Ab/Cry35Ab and Cry1F did not have insecticidal activity against this insect [6], [7].

Nowadays much research is being carried out to elucidate the molecular basis of Bt Cry toxins entomopathogenic action. The most extensively studied insecticidal Bt proteins are Cry-3D toxins and although their mode of action is not completely understood, it is generally accepted that involves toxin solubilization in the midgut of the susceptible larvae, membrane receptor binding and oligomerization of the toxin followed by pore formation in the brush border membrane [8]. Potential evidence for cell-death signalling pathways in insects as a result of Bt toxins activity has also been reported [9], [10]. However, the present knowledge about toxin-induced cellular phenomena lags behind our understanding of the physiological process of Bt intoxication. In nematodes, various signaling pathways have been involved in Cry toxicity and defensive host responses, which include p38 mitogen-activated protein kinase [11], unfolded protein response [12], DAF-2 insulin/IGFR signaling pathways [13] and hypoxia response pathways [14]. Regarding insects, several reports have characterized some of the defensive response of insects to Cry toxins by means of substraction hybridization libraries in *Choristoneura fumiferana* and *Manduca sexta* larvae treated with sublethal concentrations of Cry1Ab toxin [15], [16], transcriptional analysis in *Diabrotica virgifera* challenged with Cry3Bb toxin [17], gene silencing in *M. sexta* challenged with Cry1Ab toxin and *Aedes aegypti* intoxicated with Cry11Aa spore-crystal preparations [18], transcriptome profiling in *Tenebrio molitor* larvae after ingestion of Cry3Aa toxin [19], and proteome analyses in *Helicoverpa armigera* intoxicated with Cry1Ac [20] and *A. aegypti* exposed to Cry11Aa toxin [21], using 2D-electrophoresis and mass spectrometry.

An alternative for the analysis of proteins in a global manner is the iTRAQ technique, a powerful proteomics method that provides higher coverage than other strategies, which has been scarcely used to evaluate the physiological importance of proteins related with the Bt mode of action since only two reports have used iTRAQ to analyze differential protein alterations associated with Bt resistance [22], [23].

Traditional insect pest control methods used for stored-grain products are based on synthetic chemical pesticides that are not IPM compatible and contribute to contamination of food products constituting a risk for workers and consumers. Therefore, chemical free or biologically based approaches to control stored-product insects that have proven efficacy need to be developed [4]. Bt represents a useful alternative to conventional insecticides, formulated in bioinsecticides or delivered in transgenic plants. Bt based strategies for pest control mostly relies on Bt granular or spray surface applied products that contain mixtures of bacterial spores and insecticidal toxins. Unfortunately, currently commercialized coleopteran active Bt formulations have not proven effective against Tc and new preparations based on Bt strains more active against this insect are needed. The efficacy of the treatments might be influenced by the insect response to all components of the insecticidal formulation.

In this work, we implemented an iTRAQ proteomic analysis combined with LC-MS/MS to study the differential response of Tc larvae after intoxication with two spore-crystal mixtures of Bt strains active against this insect that produce structurally unrelated Cry toxins. Proteins differentially expressed in Bt treated larvae fell into two major categories, proteins involved in host defense and proteins linked to metabolic pathways affecting larval development, which account for both a general Bt defensive response and toxin-specific physiological regulation.

## Results and Discussion

### Bt Cry toxin producing strains display toxicity against Tc

To determine the susceptibility of Tc larvae to five coleopteran specific Bt toxin producing strains (Cry3Aa, Cry3Ba, Cry3Ca, Cry23Aa/Cry37Aa and Cry34Ab/Cry35Ab) and one lepidopteran specific Bt toxin producing strain (Cry1Ac) a single dose of 3 µg of spore-crystal mixture of each Bt toxin expressing strain per microliter of flour disc were initially assayed. In the experimental conditions, after seven days of treatment, Cry1Ac, Cry3Aa, Cry3Ca and Cry34Ab/Cry35Ab spore-crystal mixtures were not significantly active compared to non-treated larvae, whereas spore-crystal mixtures of Cry3Ba and the Tc active Cry23Aa/Cry37Aa strain [24] yielded 20±4% and 35±5% mortality, respectively (Table 1). As expected, the lepidopteran specific Cry1Ac toxin producing strain, used as a negative control, and Cry34Ab/Cry35Ab spore-crystal mixtures, already reported inactive against Tc [7], did not show toxicity. Although Cry3Aa spore-crystal mixture did not produce significant Tc larvae mortality, larval weight reduction and increased developmental time was observed in Cry3Aa treated larvae, as previously described [25].

**Table 1:** Susceptibility of Tc to Bt spore-crystal mixtures.

Treatment (3 µg of spore-crystal mixture/µL flour disc)	% Mortality (Day 7)
Cry3Aa	3±3
Cry3Ba	20±4*
Cry3Ca	0±0
Cry23Aa/Cry37Aa	35±5*
Cry34Ab/Cry35Ab	0±0
Cry1Ac	0±0
none	0±0

* Significantly different with respect to non-treated larvae (Student's *t* –test, *P*<0.05).

**Figure 1:**
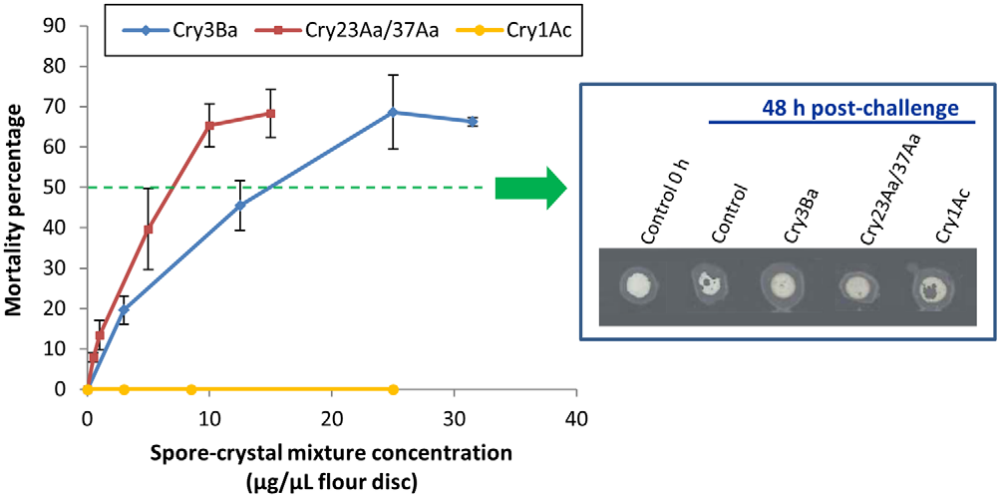
Dose-mortality assays with Cry3Ba, Cry23Aa/Cry37Aa and Cry1Ac spore-crystal mixtures in Tc larvae. Bioassays were performed on eight to ten day-old larvae fed on flour discs containing spore-crystal mixtures of Cry3Ba, Cry23Aa/Cry37Aa and Cry1Ac toxin producing Bt strains. Arrow points to images of 10 µL flour discs on which Tc larvae were fed for two days containing water (control), an approximately LC_50_ of Cry3Ba and Cry23Aa/37Aa spore-crystal preparations (12.5 µg/µL flour disc and 5.0 µg/µL flour disc, respectively), and 25 µg Cry1Ac spore-crystal mixture/µL flour disc (negative control).

Different spore-crystal mixture concentrations of Cry3Ba or Cry23Aa/Cry37Aa were assayed against Tc larvae and results are shown in Figure 1. As a negative control, a Cry1Ac spore-crystal mixture was also assayed. The profiles of Tc dose-response curves to both Bt active spore-toxin treatments were similar with a 70% maximum mortality percentage achieved after seven days of exposure, supporting that in the assay conditions, surviving larvae might be able to mount a defensive response that counteracted the toxic action. Alternatively, this maximum mortality percentage might represent the efficacy limit of the assayed treatments in this insect due to other factors, such as the insect genetic variability underlying Bt susceptibility. The LC_50_ and 95% fiducial limits calculated by Probit analysis [26] were 6.30 (4.89–8.37) µg spore-crystal mixture/µL flour disc for Cry23Aa/Cry37Aa and 13.53 (10.24–17.72) µg spore-crystal mixture/µL flour disc for Cry3Ba spore-crystal preparations. LC_50_ differences between both treatments might be due either to a differential toxic effect or to variation in the toxin content of the spore-crystal culture mixture used in the toxicity assay. Regardless of the reason, in both treatments, insects were sensing the strain active components since after two days of treatment with an LC_50_ of Cry3Ba or Cry23Aa/Cry37Aa spore-crystal mixtures, no appreciable flour disc consumption was observed evidencing cessation of feeding (Figure 1). In contrast, significant disc consumption was seen in assays with untreated larvae and larvae treated with an amount as high as 25 µg Cry1Ac spore-crystal mixture/µL flour disc (Figure 1).

### Differential protein response of Tc larvae to Bt toxin producing strains

**Figure 2:**
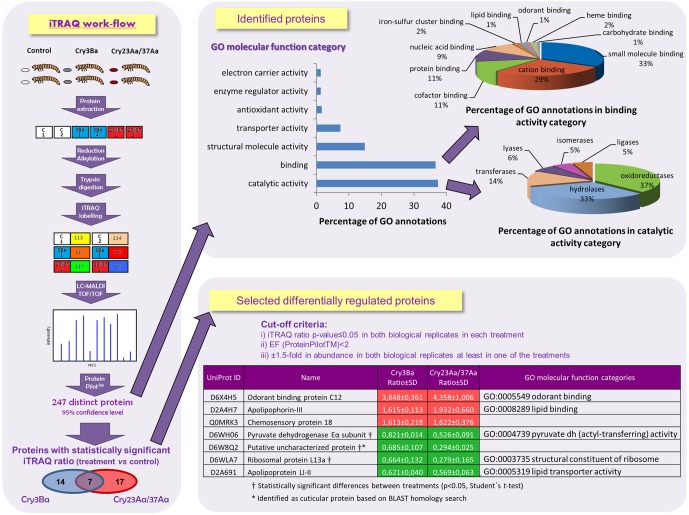
Quantitative iTRAQ proteomic analysis. A work-flow indicating the steps followed in the iTRAQ analysis performed on two biological replicates of each toxin treatment and the classification of the iTRAQ identified proteins according to GO molecular function categories are shown. Proteins showing an i-TRAQ ratio (treatment *vs* control) with a Protein Pilot p-value≤0.05 at least in one replicate sample were 21 in Cry3Ba spore-crystal treated larvae and 24 in Cry23Aa/Cry37Aa spore-crystal treated larvae. Seven differentially regulated proteins are listed for Cry3Ba and Cry23Aa/Cry37Aa treatments and were selected according to the following cut-off criteria: (i) i-TRAQ ratio p-value≤0.05 in both biological replicates in each treatment, (ii) error factor <2 (generated by ProteinPilot^TM^), (iii) an average of ±1.5-fold in abundance in response to Bt intoxication in both biological replicates at least in one of the treatments. Using Student's *t*-test, statistically significant differences between toxin treatments were detected for pyruvate dehydrogenase Eα subunit, cuticular protein and ribosomal protein L13a (p<0.05).

Toxins contained in the two spore-crystal mixtures that were shown active against Tc, Cry3Ba and Cry23Aa/Cry37Aa, belong to two different classes of Cry proteins. Cry3Ba is a Cry-3D toxin of approximately 70 kDa that lacks the C-terminal extension found in the 130 kDa Cry-3D toxins, which is dispensable for toxicity [1]. The Cry23Aa/Cry37Aa binary toxin consists of two proteins both required for toxicity, Cry23Aa toxin homologous to the dipteran active Mtx 2/3 proteins of *B. sphaericus*, and Cry37Aa toxin not related to other Bt crystal proteins [3]. Since Cry3Ba and Cry23Aa/Cry37Aa proteins are structurally unrelated and might have a distinct mode of action, we decided to analyze whether there is a differential insect response to each treatment and we undertook a proteomic approach using an iTRAQ technique combined with liquid chromatography-tandem mass spectrometry on Tc Bt treated larvae. We chose to intoxicate Tc larvae with approximately a LC_50_ dose of Cry3Ba or Cry23Aa/Cry37Aa spore-crystal mixtures to assure an active response of the insect but, in an attempt to prevent damaged gut epithelium recovery from occurring, treatments were limited to two days, when less cell damage has occurred and therefore the number of cells undergoing repair is likely reduced. Larvae were harvested right after the two days of treatment, when significant cessation of feeding was observed, well in advance of the onset of mortality, and detection of early expressed response proteins was expected. As control, larvae fed with flour discs mixed with the corresponding volume of water were used. A workflow of the iTRAQ experiment is depicted in Figure 2.

A total number of 1,669 non-redundant Tc peptide MS/MS spectra were generated, and 335 proteins were identified at a false discovery rate of 1% with PSPEP (ProteinPilot^TM^ software, ABSciex). The number of assembled proteins accurately quantified was 247 and 239, in response to Cry3Ba or Cry23Aa/Cry37Aa spore-crystal treatments, respectively (Table S1), among which 239 proteins were overlapped between the two treatments. Of the total number of 247 non-redundant identified proteins, 19.4% were identified with more than 5 peptides (Figure S1). Eighty three per cent of total identified proteins were classified according to GO molecular function categories (Figure 2). The largest GO molecular function category was catalytic activity represented mostly by oxidorreductases and hydrolases, followed by binding category, where small molecule and cation binding proteins were mainly found.

To analyze the differentially expressed proteins in response to spore-crystal treatments, the i-TRAQ ratios (treatment versus control) of the quantified proteins with a p-value≤0.05 were selected. Twenty-one proteins were found with statistically significant ratios at least in one replicate in Cry3Ba treated samples, of which 11 proteins exhibited a relative change in protein ratio below −1.5-fold or above 1.5-fold (4 out of the 11 proteins showed ±1.5-fold change in both replicate samples). In Cry23Aa/Cry37Aa treated samples, 24 proteins showed statistically significant ratios at least in one replicate, of which 20 proteins exhibited a relative change in protein ratio of ±1.5-fold (7 out of the 20 proteins showed ±1.5-fold change in both replicate samples). The 4 proteins found in Cry3Ba treated samples that showed a 1.5-fold change in both replicate samples were common to 4 of the 7 proteins found for Cry23Aa/Cry37Aa treatment that showed 1.5-fold differential expression for both of the two replicate samples. In the comparative analysis between treatments, differentially expressed proteins were selected based on the following cut-off criteria: (i) p-value≤0.05 in both biological replicates in each treatment, (ii) error factor <2 (generated by ProteinPilot^TM^), (iii) an average of at least ±1.5-fold in abundance in response to Bt intoxication in both replicates at least in one of the treatments. Seven Tc proteins (15.6% of total number of selected proteins with i-TRAQ ratios with a p-value≤0.05) showed a statistically significant change; the specific proteins and their expression alterations (fold change compared to control mass tag levels) are listed for the Cry3Ba and Cry23Aa/Cry37Aa treatments, respectively (Figure 2). Three out of these seven proteins (odorant binding protein C12, apolipophorin-III and chemosensory protein 18) were increased and showed +1.5-fold change in both replicate samples for both treatments, and four out of the seven proteins (pyruvate dehydrogenase Eα subunit, cuticular protein, ribosomal protein L13a and apolipoprotein LI-II) were decreased, when larvae treated with either of the two Bt spore-crystal mixtures were compared with non-treated control larvae. Apolipoprotein LI-II showed −1.5-fold change in both replicate samples for both treatments whereas pyruvate dehydrogenase Eα subunit, cuticular protein and ribosomal protein L13a showed a fold change smaller than 1.5 in Cry3Ba spore-crystal treated larvae that was significantly different to the 1.5-fold change reduction observed in Cry23Aa/Cry37Aa spore-crystal treated larvae (p<0.05, Student's *t*-test). In the following sections, we contend that Tc might respond to challenge by two Cry-3D toxin-spore mixtures by altering expression of proteins involved in host defense and or developmental pathways.

### Tc differentially increased proteins are likely to be implicated in host defense

Among the iTRAQ differentially increased proteins, the odorant binding protein (OBP) showed a remarkable 4-fold increase in both treatments, whereas a moderate increase of around 1.6-fold was seen for apolipophorin III and chemosensory protein 18, when comparing to untreated controls (Figure 2).

Besides its function in lipid transport, apolipophorin III has also been reported to mediate insect immune responses in several species such as *Galeria mellonella*, *Hyphantria cunea*, *Heliothis virescens*, *Locusta migratoria* and *Anopheles gambiae* [27]. In the case of coleopteran insects, apolipophorin-III gene was described to be up-regulated after larval exposure to Cry3Aa toxin in *T. molitor* [19]. Chemosensory proteins and OBP participate in sensing odors and/or pheromones [28] and have also been shown to be induced by microbial infections leading some authors to suggest a link between the olfactory system and the immune system in invertebrates [29], [30], [31].

Interestingly, in the increased protein group no significant differences between Cry3Ba and Cry23Aa/Cry37Aa Tc larvae treatments were observed (p>0.05, Student's *t*-test), indicating that induction of the corresponding genes might be a common mechanism after Bt infection that might constitute the main early defensive response of the insect, regardless of the actual process by which these two different Bt spore-crystal mixtures target cells. As OBP showed the highest change in protein levels after treatments and its role in immunity is not well established we selected this protein to further analyze its involvement in Tc host defense.

**Figure 3:**
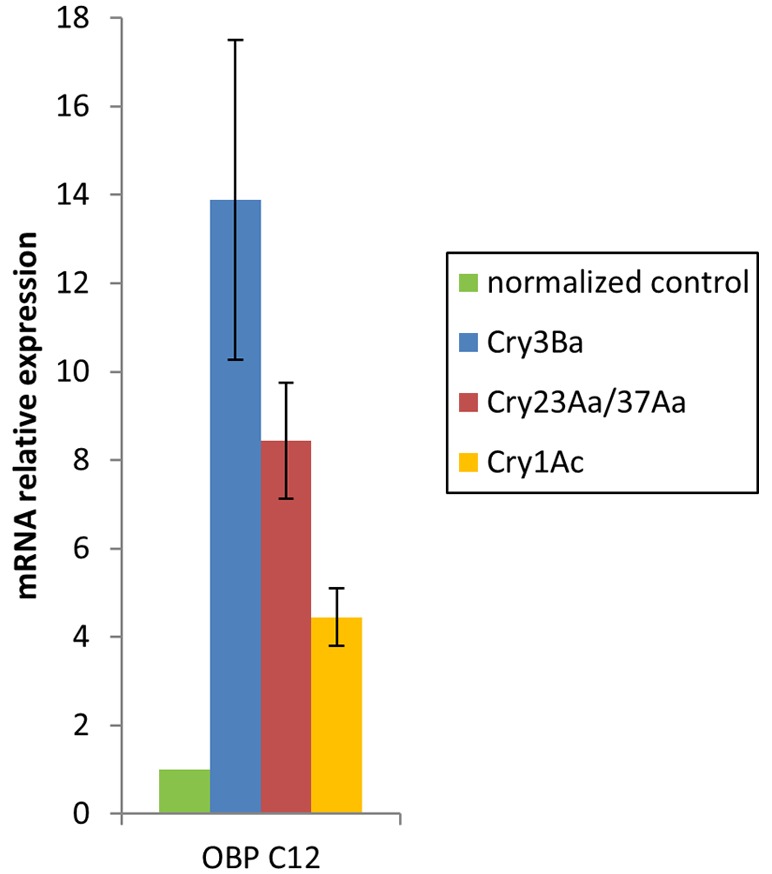
Tc OBP transcriptional analysis. qRT-PCR analysis of mRNA expression levels of Tc OBP in larvae exposed to Cry3Ba, Cry23Aa/Cry37Aa and Cry1Ac spore-crystal mixtures relative control larvae, normalized to the RPS18 mRNA. Error bars indicate standard errors of the means from two biological replicates of twenty-four individuals per replicate.

To validate at the transcription level the increased OBP protein change detected in the iTRAQ analysis, we used quantitative real time PCR (qRT-PCR) to compare mRNA expression in untreated control larvae and larvae treated with the Cry3Ba and Cry23Aa/Cry37Aa active spore-crystal mixtures or the non-toxic spore-crystal mixtures of the Cry1Ac strain (Figure 3). Results show that the OBP gene was differentially expressed after the three treatments relative to control larvae (12.4-fold, 8.0-fold and 4.5-fold up-regulation corresponding to Cry3Ba, Cry23Aa/Cry37Aa and Cry1Ac treatments, respectively). Non-significant differences between the amount of OBP transcript induced by Cry3Ba and Cry23Aa/Cry37Aa treatments were observed (p>0.05, Student's *t*-test). However, statistically significant differences between OBP transcript levels induced by any of these treatments and those induced by Cry1Ac spore-crystal treatment were found (p<0.05, Student's *t*-test). Results suggest that although OBP gene might be induced as a result of a general host defense response in Tc, its induction may be even more enhanced in response to Tc active Cry spore-crystal mixtures and therefore, this OBP protein might play a role in Tc response to Bt intoxication.

**Figure 4:**
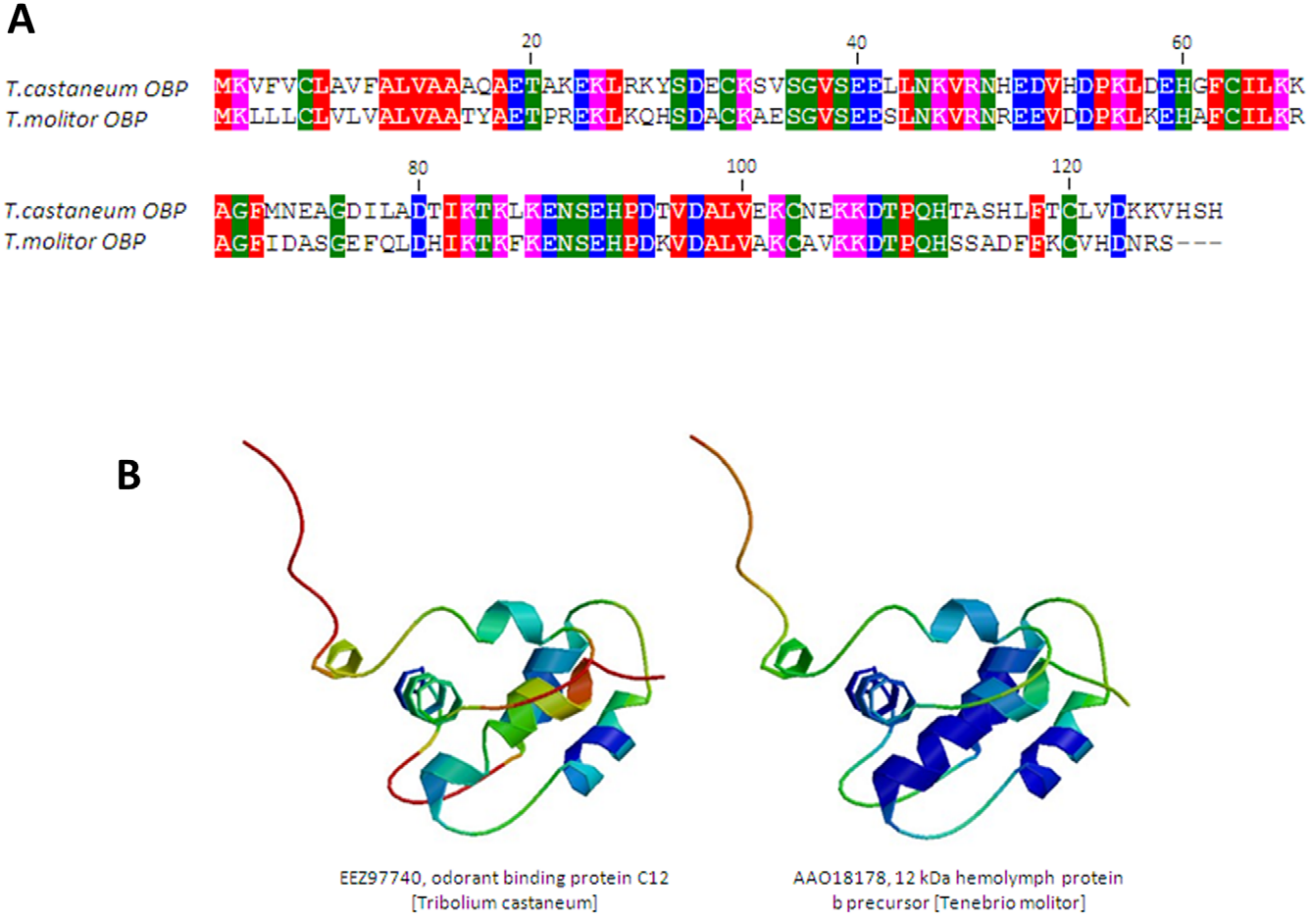
Tc OBP protein structural features. (**A**) Sequence alignment of the OBP amino acid sequences of Tc and Tm using Clustal omega [56]. (**B**) Predicted three-dimensional homology model of the Tc OBP protein using SWISS-MODEL Workspace server [32] based on Tm THP12 (1C3Y.pdb). The Tm THP12 fold (1C3Y.pdb) is shown for comparison.

A search of NCBI protein database was carried out to find sequences similar to Tc OBP (NCBI accession no. EEZ97740). *T. molitor* 12 kDa hemolymph protein b precursor (Tm THP12), characterized as an odorant binding protein in this insect [32], displayed the highest similarity (64% amino acid sequence identity). The amino acid sequence alignment of both proteins is shown in Figure 4A. Both proteins are similar to other members of the insect OBP family, particularly in structurally important regions, carrying an N-terminal signal sequence (predicted cleavage site between positions 18 and 19 using SignalP 4.0 [33], and containing four out of the six aligned cysteine residues that are diagnostic of insect OBPs [34], as described for other hemolymph OBPs [32].

We have predicted the three dimensional homology model of Tc OBP using the automated comparative protein modeling server SWISS MODEL Workspace [35], based on the solved structure of the Tm THP12 OBP (PDB accession no. 1C3Y). Consistent with the idea that both proteins share the same fold (Figure 4B), the model exhibits the same overall structure as Tm THP12 (QMEAN4 score of 0.569, Z-score of −2.26) (Figure S2).

**Figure 5:**
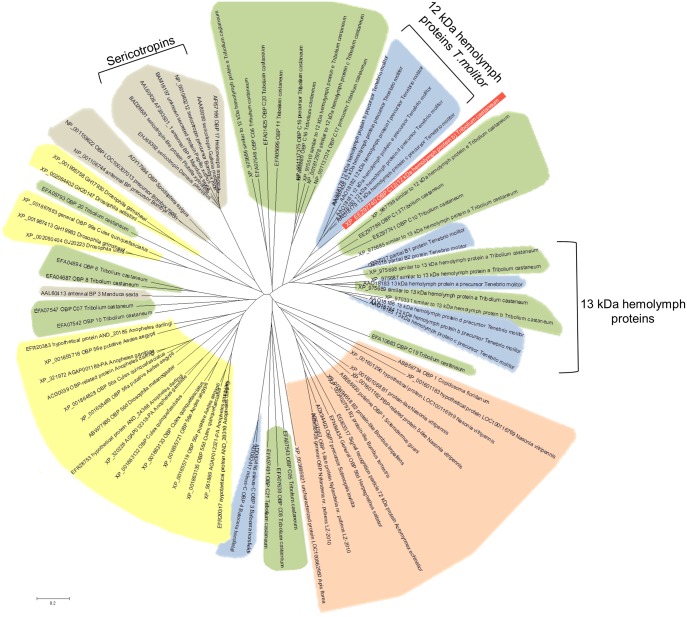
Unrooted phylogenetic tree of insect OBP proteins. The phylogenetic reconstruction was generated with MEGA version 4.0.2 software [35]. In red is shown the Tc OBP up-regulated upon Cry3Ba and Cry23Aa/Cry37Aa treatment challenge and in green, homologous Tc OBPs identified searching the BeetleBase protein database. Depicted in blue are other coleopteran OBPs, in grey lepidopteran OBPs, in yellow dipteran OBPs and in pink hymenopteran OBPs, which were identified to show homology to Tc OBP by searching the NCBI protein database.

A phylogenetic tree was inferred by the Neighbor joining method with a Gonnet matrix-based model by using Mega software [36] (Figure 5). The Tc OBP protein identified to be increased in this work clustered together with *T. molitor* 12 kDa hemolymph OBPs, indicating that Tc OBP is more similar to Tm THP12 OBP than to any of the 265 apparently functional OBPs annotated in the Tc genome [37]. It has been hypothesized that Tm THP12 and putative orthologs might be carriers of a number of small hydrophobic compounds that would normally be transported through the hemolymph [38]. Expression of OBP genes in non-olfactory tissues might indicate that the encoded proteins are likely to have non-olfactory physiological functions [39].

**Figure 6:**
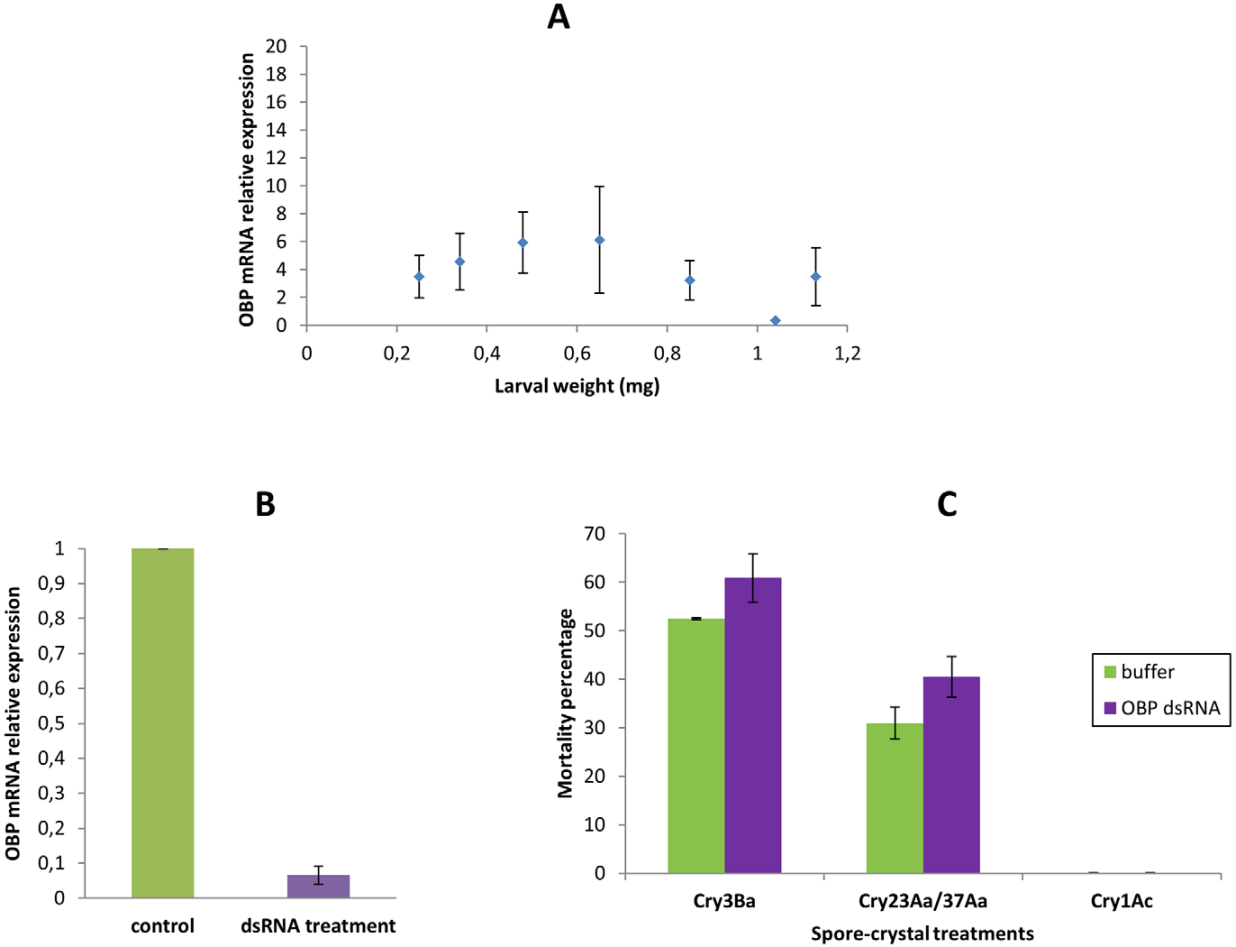
Tc OBP functional characterization. (**A**) qRT-PCR analysis of the OBP mRNA expression levels in Tc larvae of different weight, using RPS18 mRNA as reference. (**B**) OBP silencing in Tc larvae in response to injection of dsRNA against Tc OBP. The relative amount of Tc OBP transcript estimated by qRT-PCR in control and silenced larvae was compared normalized to the expression of *RPS18* gene. The statistical significance of the gene expression between the two samples was evaluated using Student's *t*-test and significant knockdown was observed (p<0.05). Error bars represent standard error of the mean of two biological replicates. (**C**) Mortality percentage following Cry3Ba, Cry23Aa/Cry37Aa or Cry1Ac spore-crystal treatments on either buffer-injected (control) or OBP dsRNA-injected larvae. Mortality experiments were performed using fifty Tc larvae in each spore-crystal treatment and the corresponding controls. Error bars represent standard error of the mean of at least two biological replicates. Mortality increase observed upon Bt treatments in OBP silenced larvae respect to buffer-injected larvae was statistically significant using Student's *t*-test (p<0.05).

In order to assess whether Tc OBP is involved in insect defense against Bt, its expression was knocked down by means of RNAi. To choose the appropriate larval size for silencing, we obtained the transcription profile of the OBP gene in different Tc larval developmental stages using qRT-PCR (Figure 6A), with RPS18 mRNA as internal control. The abundance of OBP transcripts did not significantly change in larvae of a weight range of 0.25 to 1.15 mg, except for a reduced transcript expression observed in 1.0 mg weight larvae. Since the OBP expression profile remained stable in larvae less than 1.0 mg weight, we selected larvae of 0.2 to 0.4 mg weight for silencing assays. We employed qRT-PCR to examine mRNA levels of Tc OBP in dsRNA-injected larvae and control (buffer-injected) larvae and the analysis showed a 93% reduction of Tc OBP transcript compared to control larvae (Figure 6B). Knockdown of the Tc OBP genés transcripts did not induce larval mortality, suggesting that the genés normal expression must not be essential for Tc larvae viability.

We next carried out toxicity assays with Cry3Ba, Cry23Aa/Cry37Aa or Cry1Ac spore-crystal mixtures on OBP dsRNA-injected and buffer-injected control larvae (Figure 6C). Results showed a significant increase in mortality of dsRNA-injected larvae treated either with Cry3Ba or Cry23Aa/Cry37Aa (p<0.05, Student's *t*-test), consistent with the proposed immune defense function of non-olfactory OBPs induced upon pathogen challenge [29], [30], [31]. The non-toxic Cry1Ac spore-crystal mixture treatment did not produce larval mortality neither in dsRNA-injected larvae nor in buffer-injected control larvae (Figure 6C). Although OBP gene induction was higher in Cry3Ba than in Cry23Aa/Cry37Aa treated larvae, differences observed in mRNA abundance were not statistically significant (Figure 3) and accordingly, in OBP silenced larvae mortality increase compared to buffer-injected control larvae was the same (around 10% increase) in both Cry3Ba and Cry23Aa/Cry37Aa spore-crystal treatments (Figure 6C). Results support OBP as a Tc responsive protein to Bt challenge that together with other gene products might hold a primary defense function. Understanding the effect of other host defense genes involved in Tc susceptibility to Bt will allow estimating the relative contribution of this specific OBP gene to the overall insect host defense.

### Tc differentially reduced proteins are related to metabolism and development

iTRAQ down-regulated proteins were found linked to metabolic pathways that might affect larval development. In a comparison of the protein decrease observed in Cry3Ba and Cry23Aa/Cry37Aa treatments, three out of the four down-regulated proteins showed significant differences between treatments (p<0.05, Student's *t*-test), pyruvate dehydrogenase Eα subunit, cuticular protein and ribosomal protein L13a (Figure 2). In contrast, apolipoprotein LI-II precursor protein, the common precursor of apolipoprotein I and apolipoprotein II that constitutes the basic structure of the major insect hemolymph lipophorin, showed a similar 0.6 ratio decrease in Tc larvae for both treatments. Insect lipophorin plays important roles in transporting dietary lipids from the gut to the storage depot, the fat body, while also distributing stored or biosynthesized lipids to peripheral tissues [40]. It has been reported that there is a trade-off between immune stimulation and expression of storage protein genes upon bacterial challenge [41], [42]. Interestingly, in contrast to the apolipoprotein LI-LII precursor reduction, we have observed that the immune-related apolipoprotein III protein increased, which is consistent with the reported down-regulation of accumulation of storage proteins as a consequence of activation of the immune system, considered a general strategy to redirect resources to combat injury or infection [42].

The three down-regulated proteins that showed significant differences between Cry3Ba and Cry23Aa/Cry37Aa treatments might be relevant to understand whether there is a differential insect response to each spore-crystal treatment.

Ribosomal protein L13a is a noncanonical ribosomal protein that carries out tasks often unrelated to the protein synthesis of the ribosome [43]. It has been described that regulated release of L13a from the 60S ribosomal subunit is a mechanism of transcript-specific translational control of genes involved in inflammatory processes in mammals [44]. In yeast it has been demonstrated that inactivation of the two yeast L13a homologous resulted in severe growth retardation and cell death [45]. Although the function of ribosomal protein L13a in insects is unknown, the reduced protein levels detected in the iTRAQ analysis would support a role in the midgut paralysis and cessation of feeding that characterizes Bt intoxication.

Pyruvate dehydrogenase (Eα) is the first component of the pyruvate dehydrogenase complex (PDC), responsible for the decarboxylation of pyruvate to acetyl-CoA in the mitochondria matrix, after which the acetyl-CoA enters the citric acid cycle [46]. The ability to arrest development and metabolism to cope with environmental challenges improves the survival of many species and it has been described that a hypometabolic state is characterized by suppression of oxidative pathways of energy production, involving PDC down-regulation [47]. In *Caernohabditis elegans* it has been reported that resistance to Cry pore-forming toxins can be achieved by mutations that up-regulate the hypoxia response mediated by the hypoxia inducible factor 1 (HIF-1) [14], which induces the expression of pyruvate dehydrogenase kinase that in turn inhibits PDC, leading to a suppression of mitochondrial oxidative phosphorylation. Accordingly, in Tc, pyruvate dehydrogenase Eα subunit reduction after challenging with Cry3Ba and Cry23Aa/Cry37Aa preparations would not be unexpected if it were part of the insect response to protect itself from pore-forming toxins.

The cuticle is a dynamic structure that responds to external factors such as insecticides and desiccation [48]. It is conceivable that Bt intoxication might have consequences for the expression of genes underlying cuticular functions as a part of the metabolic arrest response, affecting timing of moulting and distribution of developmental stages. In Colorado potato beetle the transition from a state of high metabolic rates and active feeding to very low metabolic rates, no feeding, and very little movement when the beetle enters diapause is characterized by a differential regulation of cuticular protein transcripts [49], [50].

**Figure 7:**
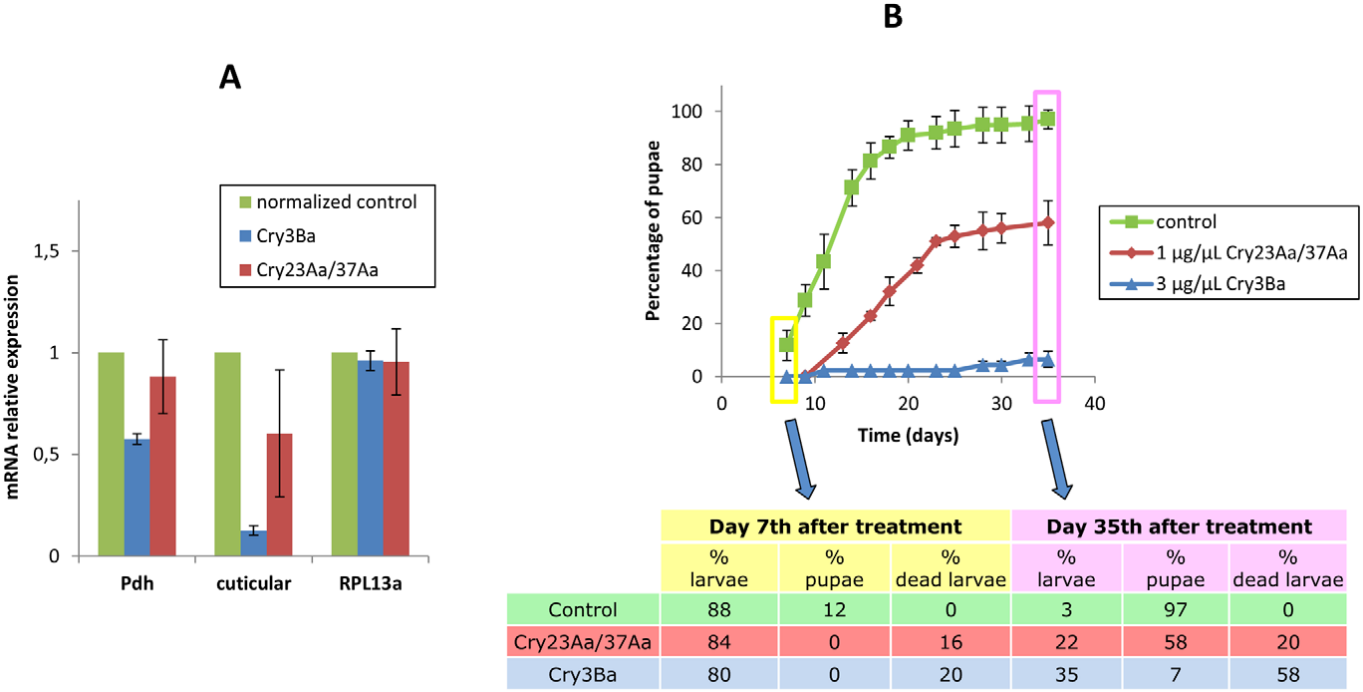
Tc differential response to Cry3Ba and Cry23Aa/Cry37Aa spore-crystal treatments. (**A**) Relative levels of Tc pyruvate dehydrogenase (Pdh), cuticular protein and ribosomal protein L13a (RPL13a) mRNAs normalized to the corresponding untreated control, determined by qRT-PCR analysis in control larvae and larvae exposed to Cry3Ba and Cry23Aa/Cry37Aa spore-crystal mixtures, using RPS18 mRNA as reference. Error bars indicate standard errors of the means from two biological replicates of twenty-four individuals per replicate. (**B**) Monitoring of Tc larvae development following Cry3Ba and Cry23Aa/Cry37Aa spore-crystal treatments that produced 15–20% mortality. Percentage of pupae was recorded after seven days of spore-crystal treatment and up to day 35, once surviving treated larvae were transferred to spore-crystal-free diet.

To gain more insight into the functional significance of the differential reduction of pyruvate dehydrogenase Eα subunit, cuticular protein and ribosomal protein L13a in CryBa and Cry23Aa/Cry37Aa treatments, expression of the corresponding genes was further assessed at the transcription level using qRT-PCR in untreated control larvae and intoxicated larvae (Figure 7A). For ribosomal protein L13a, no significant differences in gene expression were observed in any of the toxin treatments with respect to non-treated control larvae. For pyruvate dehydrogenase Eα subunit and cuticular protein significant differences between mRNA abundance in treated and control larvae were only found in Cry3Ba treatment. Intriguingly, at protein level both spore-crystal treatments led to down-regulation of the two proteins and a significant higher reduction was observed in Cry23Aa/Cry37Aa intoxicated larvae. These results suggest a distinct transcriptional regulation depending upon the type of Bt toxin used in larval treatment. As these two proteins might be involved in metabolic and developmental processes, differential transcriptional regulation might influence how larvae recover from Bt challenge. Therefore, we decided to intoxicate Tc larvae with a dose of Cry3Ba or Cry23Aa/Cry37Aa spore-crystal mixtures causing 15–20% mortality after 7 days of toxin treatment to assure low insect mortality and then surviving larvae were fed flour discs without toxin to let the gut epithelium recover from toxin damage. Figure 7B shows that the pupation profile of surviving larvae in Cry23Aa/Cry37Aa treatment was similar to that of non-treated control larvae although the pupation rate was reduced for Cry23Aa/Cry37Aa treated larvae, reaching a maximum of 58% pupation (relative to pupation percentage in control larvae) on day 35^th^ after the initial treatment. In contrast, on that day, only 7% of Cry3Ba surviving larvae reached pupation. Additionally, differences between Cry3Ba and Cry23Aa/Cry37Aa treatments were observed regarding larval mortality on day 35^th^ after the initial treatment, being three times higher for Cry3Ba than for Cry23Aa/Cry37Aa intoxicated larvae. The larval developmental arrest observed in Cry3Ba surviving larvae as opposed to the 60% of Cry23Aa/Cry37Aa surviving larvae that recovered from toxin treatment and reached pupation is in agreement with the transcriptional repression of pyruvate dehydrogenase and the cuticular protein genes observed only after Cry3Ba treatment (Figure 7A). Other coleopteran active toxins structurally related to Cry3Ba, also altered transcription of genes linked to metabolic processes. Cry3Bb toxin has been reported to impact larval metabolism and development in *D. virgifera* [17], and Cry3Aa toxin exposure resulted in a repression of genes encoding metabolic enzymes associated with proteolysis, glycolysis, TCA, and fatty acid metabolism in *T. molitor* [19].

## Conclusions

The use of iTRAQ combined with LC-MS/MS has led to the discovery of several novel differentially expressed proteins in Tc larvae in response to spore-crystal mixtures of Bt strains producing two structurally unrelated Cry toxins, Cry3Ba and Cry23Aa/Cry37Aa. Tc larvae early response involved up-regulation of three host defense related proteins (odorant binding protein C12, apolipophorin-III and chemosensory protein 18) and down-regulation of four proteins that play a role in larval metabolism and development (pyruvate dehydrogenase Eα subunit, cuticular protein, ribosomal protein L13a and apolipoprotein LI-II). At the transcriptional level, pyruvate dehydrogenase and cuticular genes were decreased in Tc larvae exposed to the Cry3Ba producing strain compared to the Cry23Aa/Cry37Aa producing strain, which may contribute to the developmental arrest that we observed with larvae fed the Cry3Ba producing strain.

Better understanding how target insects respond to Bt intoxication will allow improving Bt strategies for pest control and counteracting resistance development.

## Materials and Methods

### Toxin production

Cry3Aa, Cry3Ba, Cry3Ca and Cry1Ac crystals were produced in Bt strains BTS1, BTS00125L, BTS02109P and HD73, respectively. The binary toxins Cry23Aa/Cry37Aa and Cry34Ab/Cry35Ab were produced in Bt strains EG10327 (Ref. No. NRRL B-21365) and PS149B1 (Ref. No. NRRL B-21553), respectively, obtained from the Agricultural Research Culture Collection, Northern Regional Research Laboratory (NRRL), USA.

All bacterial strains were grown in solid sporulation medium [51] at 30±1°C until complete autolysis. Lysed bacteria were resuspended in 2x PBS pH 7.4 and washed twice with 0.02% Triton X-100 in 2x PBS pH 7.4 and twice with water. Following centrifugation at 6,000x *g* for 10 min at 4°C, spore-crystal mixtures were resuspended in water and stored at −20°C until use.

### Insects

A laboratory colony of Tc founded from Ga-2 strain adults kindly provided by Dr. Beeman (USDA) was used. Insects were reared on whole-grain flour with 5% brewer yeast powder at 30±1°C in darkness.

### Toxicity assays

Toxicity assays were performed on Tc larvae eight to ten days old after egg eclosion, fed for seven days on 20 µL flour discs (20% flour, w/v), prepared as in [52], containing 3 µg Cry3Aa, Cry3Ba, Cry3Ca, Cry23Aa/Cry37Aa, Cry34Ab/Cry35Ab and Cry1Ac spore-crystal mixtures per microliter of flour disc for treatments or water, in control assays. Assays were performed in 96-well polystyrene plates (Sterilin, Thermofisher) with one flour disc and one larva per well. Twenty-four larvae were assayed for each toxin and at least three replicates were carried out. Mortality was recorded after 7 days under laboratory rearing conditions. Cry3Ba and Cry23Aa/Cry37Aa LC_50_ were estimated from several spore-crystal mixture doses using Probit analysis [26].

For toxicity assays on silenced larvae, four days after dsRNA injection, larvae were exposed to 12.5 µg/µL Cry3Ba or 5.0 µg/µL Cry23Aa/Cry37Aa spore-crystal mixtures in flour discs. Flour discs prepared with water were used as controls. At least two replicates of fifty larvae were assayed for each toxin and mortality was recorded after 7 days under laboratory rearing conditions.

### iTRAQ Analysis

Forty Tc eight-day-old larvae (0.38±0.01 mg) treated with 12.5 µg Cry3Ba spore-crystal mixture/µL flour disc or 5.0 µg Cry23Aa/Cry37Aa spore-crystal mixture/µL flour disc for two days, and untreated control larvae were used to prepare protein extracts from whole larvae in 4 M urea pH 7.4, 0.05% Protease Max surfactant (Promega), 48 μM pepstatin A, 20 μM E-64. Following centrifugation at 10,000x *g* for 10 min at 4°C, the supernatant was separated, protein concentration measured by the protein-dye method of Bradford [53] using BSA as a standard and stored at −80°C.

Two independent biological replicates of toxin treated or control larvae were labeled with i113, i114, i115, i116, i117, i118 iTRAQ reagents according to the manufacturer's protocol (Applied Biosystems). The tag labeling order was untreated control replicate-1 113; untreated control replicate-2 114; Cry3Ba spore-crystal mixture treated replicate-1 115; Cry3Ba spore-crystal mixture treated replicate-2 116; Cry23Aa/Cry37Aa spore-crystal mixture treated replicate-1 117; Cry23Aa/Cry37Aa spore-crystal mixture treated replicate-2 118. Labelled protein samples, reduced and alkylated, were digested using trypsin. The resulting labelled peptides were then pooled for further processing and analyzed by integrated LC and MALDI-TOF/TOF analyzer QSTAR ESI. MS/MS spectra were analyzed using the Paragon algorithm in ProteinPilot^TM^ software (ABSciex) with the default search program with digestion enzyme trypsin and methyl methanethiosulfonate as cysteine modification. Data was normalized for loading error by bias correction calculated with ProGroup algorithm and to reduce false positive identification results, a minimum unused ProtScore of 1.3 equivalent to 95% confidence and false discovery rate (FDR) less than 1% were required for all reported proteins. A protein was considered significantly identified when one or more high-confidence (>95%) unique peptides were assigned and the iTRAQ quantification fold difference *p*-value was <0.05. The protein search was performed against NCBI protein Tc database. Gene Ontology (GO) terms were retrieved from UniProt database (http://www.uniprot.org).

### RNA isolation and cDNA synthesis

Total RNA was isolated from Tc larvae using Trizol LS Reagent (Invitrogen), following the manufacturer's protocol, and the purified RNA was treated with DNase I (DNA-free, Ambion, Inc.). The ratio of absorbance at 260 nm to 280 nm (A_260_/A_280_ ratio) was used to assess the purity of RNA samples and RNA quality was evaluated by 1% agarose gel electrophoresis and quantified spectrophotometrically (NanoDrop 2000, Thermo Scientific). AMV reverse transcriptase (2 U/µL, final concentration) (Roche) was employed for first strand cDNA synthesis using 1 µg RNA, 50 ng/µL oligo (dT)_15_ (Promega) and 2.5 µM random hexamers (Applied Biosystems).

### Quantitative real-time PCR (qRT-PCR)

qRT-PCR was performed on each template in a final volume of 25 µL using 100 ng on a StepOnePlus Real-Time PCR system (Applied Biosystems) thermocycler, following the manufacturer's recommendations, using Power SYBR Green PCR Master Mix (Applied Biosystems). Gene specific forward and reverse primers (Table S2) were designed with Primer Express software (Applied Biosystems) using sequences retrieved from BeetleBase website (http://www.Beetlebase.org) [54] under the following accession numbers TC011411-RA (OBP C12 protein), TC002948-RA (pyruvate dehydrogenase protein), TC013477-RA (ribosomal protein L13a) and TC000726-RA (cuticular protein). Primers were obtained from IDT Technologies and optimization of primer concentration was performed using 10 ng cDNA per reaction. Single, sharply defined melting curves confirmed the specificity of the chosen primers, as well as minimization of primer-dimers formation. The primer concentrations used are included in Table S2. Primer effieciencies were calculated using different cDNA concentrations in the range 80–8000 pg/µL (Table S2). Amplifications were carried out using two biological replicates of cDNA, each one from RNA obtained from twenty larvae, and the mean values of three technical replicates were analyzed. RPS18 (ribosomal protein S18) gene (Accession number TC014405-RA), reported as a stable reference gene for qRT-PCR in TC [55], was used to normalize gene expression (forward and reverse primer sequences included in Table S2). Gene expression relative-fold was calculated with the comparative C_t_ (ΔΔC_t_) method, using the StepOne software (Applied Biosystems). Data were analyzed by Student's *t*-test for statistically significant differences (p<0.05).

To analyze the expression of Tc odorant binding protein gene during larval development, quantitative real-time PCR was performed as described above on RNA obtained from larvae of different weight within a range of 0.25 to 1.2 mg.

### RNAi

RNA isolation and cDNA synthesis was performed as described above, from eight to ten day-old larvae after egg eclosion. cDNA was used as template for PCR amplification using Prime Star polymerase (Takara) and specific primers generated from Tc OBP gene sequence (NCBI accession no. EEZ97740), containing a T7 polymerase promoter sequence at their 5′ end (Table S2). PCR product (1 µg) was used for *in vitro* transcription to prepare dsRNA using Ambion MEGAscript T7 kit (Applied Biosystems) according to the manufacturer's protocol. Purified dsRNA was stored at −80°C until injected into Tc larvae.

Eight day old larvae were anaesthetized for 5 min on ice before ventral injection of approximately 0.2 µL of 1 µg/µL dsRNA in injection buffer (1.4 mM NaCl, 0.07 mM Na_2_HPO_4_, 0.03 mM KH_2_PO_4_, 4 mM KCl), using thin wall capillars (World Precision Instruments) in a microinjection system (Narishige). Control larvae were injected with buffer. Following injection, larvae were grown under standard rearing conditions.

In OBP silenced larvae, OBP transcript levels were evaluated 8 days after dsRNA injection by qRT-PCR from total RNA, using the OBP forward and reverse primers described in Table S2.

## Supporting Information

Figure S1 **Number of peptide identifications per protein in the iTRAQ analysis.**
(TIF)

Figure S2 **Tc OBP SWISS MODEL Workspace automated model.**
(PDF)

Table S1 **Proteins identified in the iTRAQ analysis.**
(XLSX)

Table S2 **Primers used in qRT-PCR to analyze the expression of genes corresponding to iTRAQ differentially expressed proteins upon toxin treatments and to generate dsRNA in RNAi experiments.**
(TIF)

## References

[1] Schnepf E, Crickmore N, Van Rie J, Lereclus D, Baum J, et al (1998) *Bacillus thuringiensis* and its pesticidal crystal proteins. Microbiol Mol Biol Rev 62: 775–806. 9729609 10.1128/mmbr.62.3.775-806.1998 PMC98934

[2] Crickmore N, Zeigler DR, Schnepf E, van Rie J, Lereclus D, et al. (2012) *Bacillus thuringiensis* toxin nomenclature. Available: http://www.lifesci.sussex.ac.uk/Home/Neil_Crickmore/Bt/. Accessed 2012 Jul 31.

[3] de Maagd RA, Bravo A, Berry C, Crickmore N, Schnepf HE (2003) Structure, diversity, and evolution of protein toxins from spore-forming entomopathogenic bacteria. Annu Rev Genet 37: 409–433. 14616068 10.1146/annurev.genet.37.110801.143042

[4] Phillips TW, Throne JE (2010) Biorational approaches to managing stored-product insects. Annu Rev Entomol 55: 375–397. 19737083 10.1146/annurev.ento.54.110807.090451

[5] Morris K, Lorenzen MD, Hiromasa Y, Tomich JM, Oppert C (2009) *Tribolium castaneum* larval gut transcriptome and proteome: a resource for the study of the coleopteran gut. J Proteome Res 8: 3889–3898. 19545177 10.1021/pr900168z

[6] van Frankenhuyzen K, Nystrom C (2009) *The Bacillus thuringiensis* Toxin Specificity Database. Available: http://www.glfc.cfs.nrcan.gc.ca/bacillus. Accessed 31 July.

[7] Oppert B, Ellis RT, Babcock J (2010) Effects of Cry1F and Cry34Ab1/35Ab1 on storage pests. J Stored Products Res 46: 143–148.

[8] Bravo A, Gill SS, Soberon M (2007) Mode of action of *Bacillus thuringiensis* Cry and Cyt toxins and their potential for insect control. Toxicon 49: 423–435. 17198720 10.1016/j.toxicon.2006.11.022 PMC1857359

[9] Zhang X, Candas M, Griko NB, Taussig R, Bulla Jr LA (2006) A mechanism of cell death involving an adenylyl cyclase/PKA signaling pathway is induced by the Cry1Ab toxin of *Bacillus thuringiensis* . Proc Natl Acad Sci USA 103: 9897–9902. 16788061 10.1073/pnas.0604017103 PMC1502550

[10] Zhang X, Griko NB, Corona SK, Bulla Jr LA (2008) Enhanced exocytosis of the receptor BT-R1 induced by the Cry1Ab toxin of *Bacillus thuringiensis* directly correlates to the execution of cell death. Comp Biochem Physiol B 149: 581–588. 18230416 10.1016/j.cbpb.2007.12.006

[11] Huffman DL, Abrami L, Sasik R, Corbeil J, van der Goot FG, et al (2004) Mitogen-activated protein kinase pathways defend against bacterial poreforming toxins. Proc Natl Acad Sci U S A 101: 10995–11000. 15256590 10.1073/pnas.0404073101 PMC503732

[12] Bischof LJ, Kao CY, Los FC, Gonzalez MR, Shen Z, et al (2008) Activation of the unfolded protein response is required for defenses against bacterial poreforming toxin in vivo. PLoS Pathog 4: e1000176. 18846208 10.1371/journal.ppat.1000176 PMC2553261

[13] Chen CS, Bellier A, Kao CY, Yang YL, Chen HD, et al (2010) WWP-1 is a novel modulator of the DAF-2 insulin-like signaling network involved in pore-forming toxin cellular defenses in Caenorhabditis elegans. PLoS ONE 5: e9494. 20209166 10.1371/journal.pone.0009494 PMC2830483

[14] Bellier A, Chen C-S, Kao C-Y, Cinar HN, Aroian RV (2009) Hypoxia and the hypoxic response pathway protect against pore-forming toxins in *C. elegans* . PLoS Pathog 5(12): e1000689. 20011506 10.1371/journal.ppat.1000689 PMC2785477

[15] Meunier L, Préfontaine G, van Munster M, Brousseau R, Masson L (2006) Transcriptional response of *Choristoneura fumiferana* to sublethal exposure of Cry1Ab protoxin from *Bacillus thuringiensis* . Insect Mol Biol 15: 475–483. 16907834 10.1111/j.1365-2583.2006.00659.x

[16] van Munster M, Préfontaine G, Meunier L, Elias M, Mazza A, et al (2007) Altered gene expression in *Choristoneura fumiferana* and *Manduca sexta* in response to sublethal intoxication by *Bacillus thuringiensis* Cry1Ab toxin. Insect Mol Biol 16: 25–35. 17257206 10.1111/j.1365-2583.2006.00692.x

[17] Sayed A, Wiechman B, Struewing I, Smith M, French W, et al (2010) Isolation of transcripts from *Diabrotica virgifera virgifera* LeConte responsive to the *Bacillus thuringiensis* toxin Cry3Bb1. Insect Mol Biol 19: 381–389. 20337747 10.1111/j.1365-2583.2010.00998.x

[18] Cancino-Rodezno A, Alexander C, Villaseñor R, Pacheco S, Porta H, et al (2010) The mitogen-activated protein kinase p38 is involved in insect defense against Cry toxins from *Bacillus thuringiensis* . Insect Biochem Mol Biol 40: 58–63. 20040372 10.1016/j.ibmb.2009.12.010 PMC2827608

[19] Oppert B, Dowd SE, Bouffard P, Li L, Conesa A, et al (2012) Transcriptome profiling of the intoxication response of *Tenebrio molitor* larvae to *Bacillus thuringiensis* Cry3Aa protoxin. PLoS ONE 7(4): e34624. 22558093 10.1371/journal.pone.0034624 PMC3338813

[20] Yuan C, Ding X, Xia L, Yin J, Huang S, et al (2011) Proteomic analysis of BBMV in *Helicoverpa armigera* midgut with and without Cry1Ac toxin treatment. Biocontrol Sci Technol 21: 139–151.

[21] Cancino-Rodezno A, Lozano L, Oppert C, Castro JI, Lanz-Mendoza H, et al (2012) Comparative proteomic analysis of *Aedes aegypti* larval midgut after intoxication with Cry11Aa toxin from *Bacillus thuringiensis* . PLoS ONE 7(5): e37034. 22615881 10.1371/journal.pone.0037034 PMC3353955

[22] Tiewsiri K, Wang P (2011) Differential alteration of two aminopeptidases N associated with resistance to *Bacillus thuringiensis* toxin Cry1Ac in cabbage looper. Proc Natl Acad Sci USA 108: 14037–14042. 21844358 10.1073/pnas.1102555108 PMC3161562

[23] Zhang X, Tiewsiri K, Kain W, Huang L, Wang P (2012) Resistance of *Trichoplusia ni* to *Bacillus thuringiensis* toxin Cry1Ac is independent of alteration of the cadherin-like receptor for Cry toxins. PLoS ONE 7(5): e35991. 22606242 10.1371/journal.pone.0035991 PMC3351398

[24] Donovan WP, Slaney AC, Donovan JC (2002) *Bacillus thuringiensis* cryET33 and cryET34 compositions and uses thereof. US Patent No.549839. Monsanto Technology LLC.

[25] Oppert B, Morgan TD, Kramer KJ (2011) Efficacy of *Bacillus thuringiensis* Cry3Aa protoxin and protease inhibitors against coleopteran storage pests. Pest Manag Sci 67: 568–573. 21268232 10.1002/ps.2099

[26] Finney DJ (1947) Probit analysis; a statistical treatment of the sigmoid response curve. Oxford: Macmillan. 256 p.

[27] Gupta L, Noh JY, Jo YH, Oh SH, Kumar S, et al (2010) Apolipophorin-III mediates antiplasmodial epithelial responses in *Anopheles gambiae* (G3) mosquitoes. PLoS ONE 5(11): e15410. 21072214 10.1371/journal.pone.0015410 PMC2970580

[28] Krieger J, von Nickisch-Rosenegk E, Mameli M, Pelosi P, Breer H (1996) Binding proteins from the antennae of *Bombyx mori* . Insect Biochem Mol Biol 26: 297–307. 8900598 10.1016/0965-1748(95)00096-8

[29] Levy F, Rabel D, Charlet M, Bulet P, Hoffmann JA, et al (2004) Peptidomic and proteomic analyses of the system immune response of *Drosophila* . Biochimie 86: 607–616. 15556270 10.1016/j.biochi.2004.07.007

[30] Aguilar R, Jedlicka AE, Mintz M, Mahairaki V, Scott AL, et al (2005) Global gene expression analysis of *Anopheles gambiae* responses to microbial challenge. Insect Biochem Mol Biol 35: 709–719. 15894188 10.1016/j.ibmb.2005.02.019

[31] Song KH, Jung SJ, Seo YR, Kang SW, Han SS (2006) Identification of up-regulated proteins in the hemolymph of immunized *Bombyx mori* larvae. Comp Biochem Physiol D 1: 260–266. 10.1016/j.cbd.2006.01.001 20483257

[32] Graham LA, Tang W, Baust JG, Liou Y-C, Reid TS, et al (2001) Characterization and cloning of a *Tenebrio molitor* hemolymph protein with sequence similarity to insect odorant-binding proteins. Insect Biochem Mol Biol 31: 691–702. 11267907 10.1016/s0965-1748(00)00177-6

[33] Petersen TN, Brunak S, von Heijne G, Nielsen H (2011) SignalP 4.0: discriminating signal peptides from transmembrane regions Nat Methods 8: 785–786. Available: http://www.cbs.dtu.dk/services/SignalP/. Accessed 2013 Jan 2. 10.1038/nmeth.1701 21959131

[34] Pelosi P, Maida M (1995) Odorant-binding proteins in insects. Comp Biochem Physiol B 111: 503–514. 7613772 10.1016/0305-0491(95)00019-5

[35] Arnold K, Bordoli L, Kopp J, Schwede T (2006). The SWISS-MODEL Workspace: A web-based environment for protein structure homology modelling. Bioinformatics 22: 195–201. Available: http://swissmodel.expasy.org/workspace/. Accessed 2013 Jan 2. 10.1093/bioinformatics/bti770 16301204

[36] Tamura K, Peterson D, Peterson N, Stecher G, Nei M, et al. (2011) MEGA5: Molecular Evolutionary Genetics Analysis using maximum mikelihood, evolutionary distance, and maximum parsimony methods. Mol. Biol. Evol. 28: 2731-2739. Available: http://www.megasoftware.net. Accessed 2013 Jan 2. 10.1093/molbev/msr121 PMC3203626 21546353

[37] *Tribolium* Genome Sequencing Consortium (2008) The genome of the model beetle and pest *Tribolium castaneum* . Nature 452: 949–955. 18362917 10.1038/nature06784

[38] Graham LA, Brewer D, Lajoie G, Davies PL (2003) Characterization of a subfamily of beetle odorant-binding proteins found in hemolymph. Mol Cell Proteomics 2: 541–549. 12883044 10.1074/mcp.M300018-MCP200

[39] Armbruster P, White S, Dzundza J, Crawford J, Zhao X (2009) Identification of Genes Encoding Atypical Odorant-Binding Proteins in *Aedes albopictus* (Diptera: Culicidae). J Med Entomol 46: 271–280. 19351077 10.1603/033.046.0211

[40] Wang C, Cao Y, Zhongkang W, Yin Y, Peng G, et al (2007) Differentially-expressed glycoproteins in *Locusta migratoria* hemolymph infected with *Metarhizium anisopliae* . J Invertebr Pathol 96: 230–236. 17658547 10.1016/j.jip.2007.05.012

[41] Meng Y, Omuro S, Funaguma S, Daimon T, Kawaoka S, et al (2008) Prominent down-regulation of storage protein genes after bacterial challenge in eri-silkworm, *Samia cynthia ricini* . Arch Insect Biochem Physiol 67: 9–19. 18064702 10.1002/arch.20214

[42] Lourenço AP, Martins JR, Bitondi MMG, Simôes LP (2009) Trade-off between immune stimulation and expression of storage protein genes. Arch Insect Biochem Physiol 71: 70–87. 19309002 10.1002/arch.20301

[43] Chaudhuri S, Vyas K, Kapasi P, Komar AA, Dinman JD, et al (2007) Human ribosomal protein L13a is dispensable for canonical ribosome function but indispensable for efficient rRNA methylation. RNA 13: 2224–2237. 17921318 10.1261/rna.694007 PMC2080596

[44] Vyas K, Chaudhuri S, Leaman DW, Komar AA, Musiyenko A, et al (2009) Genome-wide polysome profiling reveals an inflammation-responsive posttranscriptional operon in gamma interferon-activated monocytes. Mol Cell Biol 29: 458–470. 19001086 10.1128/MCB.00824-08 PMC2612521

[45] Chen FW, Ioannou YA (1999) Ribosomal proteins in cell proliferation and apoptosis. Int Rev Immunol 18: 429–448. 10672495 10.3109/08830189909088492

[46] Harris RA, Bowker-Kinley MM, Huang BL, Wu PF (2002) Regulation of the activity of the pyruvate dehydrogenase complex. In: Weber G, editors. Advances in enzyme regulation. Proceedings, vol. 42: 249–259. 10.1016/s0065-2571(01)00061-9 12123719

[47] Hand SC, Menze MA, Borcar A, Patil Y, Covi JA, et al (2011) Metabolic restructuring during energy-limited states: Insights from *Artemia franciscana* embryos and other animals. J Insect Physiol 57: 584–594. 21335009 10.1016/j.jinsphys.2011.02.010 PMC3104064

[48] Zhang J, Goyer C, Pelletier Y (2008) Environmental stresses induce the expression of putative glycine-rich insect cuticular protein genes in adult *Leptinotarsa decemlineata* (Say). Insect Mol Biol 17: 209–216. 18477239 10.1111/j.1365-2583.2008.00796.x

[49] Yocum GD, Rinehart JP, Chirumamilla-Chapara A, Larson ML (2009) Characterization of gene expression patterns during the initiation and maintenance phases of diapause in the Colorado potato beetle, *Leptinotarsa decemlineata* . J Insect Physiol 55: 32–39. 18992752 10.1016/j.jinsphys.2008.10.003

[50] Yocum GD, Rinehart JP, Larson ML (2009) Down-regulation of gene expression between the diapause initiation and maintenance phases of the Colorado potato beetle, *Leptinotarsa decemlineata* (Coleoptera: Chrysomelidae). Eur J Entomol 106: 471–476.

[51] Stewart GS, Johnstone K, Hagelberg E, Ellar DJ (1981) Commitment of bacterial spores to germinate. A measure of the trigger reaction. Biochem J 198: 101–106. 6798972 10.1042/bj1980101 PMC1163215

[52] Xie YS, Bodnaryk RP, Fields PG (1996) A rapid and simple flour-disk bioassay for testing substances active against stored-product insects. Can Entomol 128: 865–875.

[53] Bradford MM (1976) A rapid and sensitive method for the quantitation of microgram quantities of protein utilizing the principle of protein-dye binding. Anal Biochem 72: 248–254. 942051 10.1016/0003-2697(76)90527-3

[54] Kim HS, Murphy T, Xia J, Caragea D, Park Y, et al (2010) BeetleBase in 2010: revisions to provide comprehensive genomic information for *Tribolium castaneum* . Nucleic Acids Res 38: D437–442. 19820115 10.1093/nar/gkp807 PMC2808946

[55] Lord JC, Hartzer K, Toutges M, Oppert B (2010) Evaluation of quantitative PCR reference genes for gene expression studies in *Tribolium castaneum* after fungal challenge. J Microbiol Methods 80: 219–221. 20026205 10.1016/j.mimet.2009.12.007

[56] Sievers F, Wilm A, Dineen DG, Gibson TJ, Karplus K, et al. (2011) Fast, scalable generation of high-quality protein multiple sequence alignments using Clustal Omega. Mol Syst Biol 7: 539. Available: http://www.ebi.ac.uk/Tools/msa/clustalo/. Accessed 2013 Jan 2. 10.1038/msb.2011.75 PMC3261699 21988835

