# Supplementary figures and images for: Proteome Response of Tribolium castaneum Larvae to Bacillus thuringiensis Toxin Producing Strains
Source: PLoS One. 2013 Jan 25;8(1):e55330. doi: 10.1371/journal.pone.0055330 (PMC3555829; doi:10.1371/journal.pone.0055330)

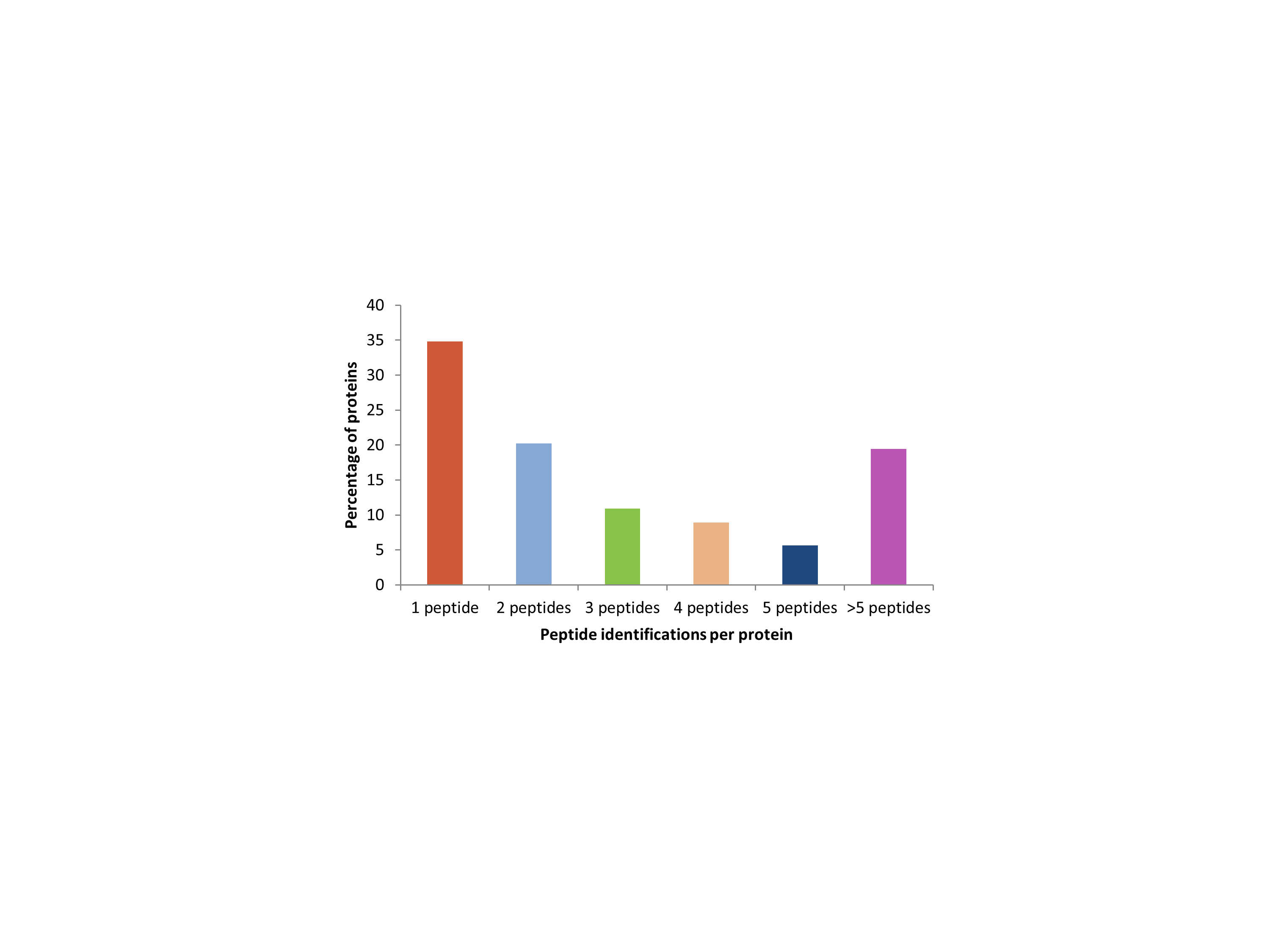

Supplement: Figure S1 — Number of peptide identifications per protein in the iTRAQ analysis. (TIF) [file pone.0055330.s001.tif]

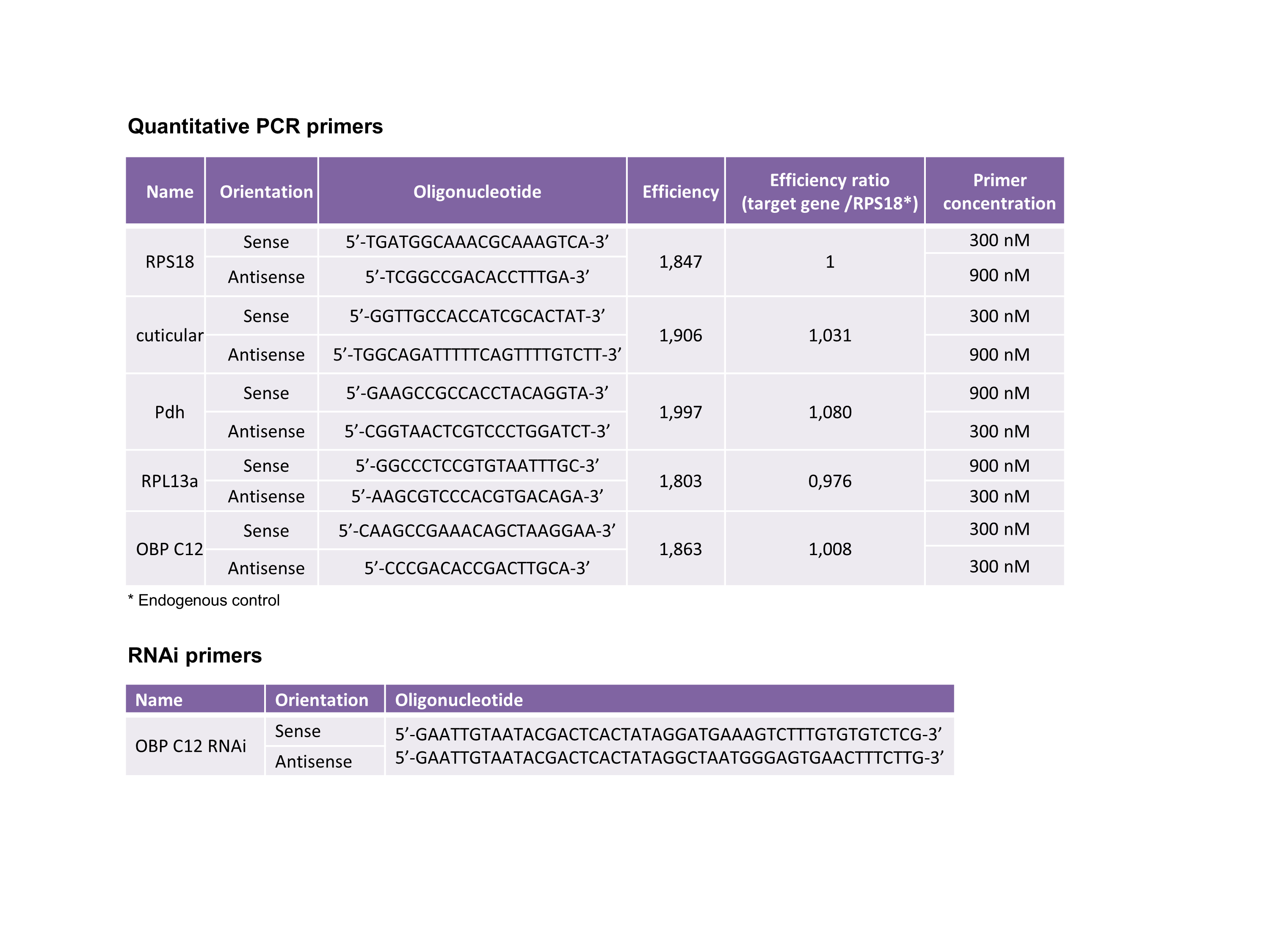

Supplement: Table S2 — Primers used in qRT-PCR to analyze the expression of genes corresponding to iTRAQ differentially expressed proteins upon toxin treatments and to generate dsRNA in RNAi experiments. (TIF) [file pone.0055330.s004.tif]
