# Supplementary material for: Proteome Response of Tribolium castaneum Larvae to Bacillus thuringiensis Toxin Producing Strains
Source: PLoS One. 2013 Jan 25;8(1):e55330. doi: 10.1371/journal.pone.0055330 (PMC3555829; doi:10.1371/journal.pone.0055330)
Supplement: Figure S2 — Tc OBP SWISS MODEL Workspace automated model. (PDF) [file pone.0055330.s002.pdf]

SWISS-MODEL Workspace

Workunit: P000003 Title:OBP

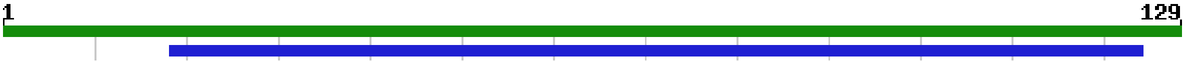

Model Summary:

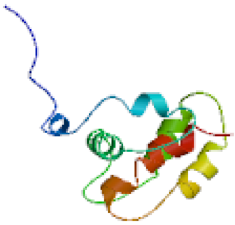

Model information:

Modelled residue range:	19 to 125
Based on template:	1c3yA (99.9 A)
Sequence Identity [%]:	61.682
Evalue:	7.96001e-28

Quality information:

QMEAN Z-Score: -2.26

Quaternary structure information:

Template (1c3y): MONOMER
Model: MONOMER

Ligand information:

Ligands in the template: none.
Ligands in the model: none.

Global Model Quality Estimation:

QMEAN4 global scores:

QMEANscore4: 0.569

Estimated absolute model quality:

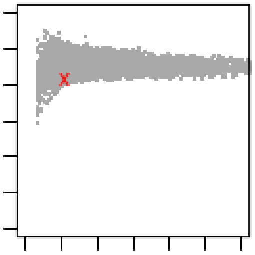

Z-Score: -2.26

Score components:

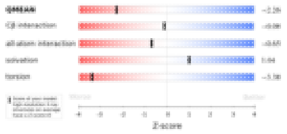

Local scores:

Coloring by residue error:

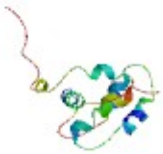

Residue error plot:

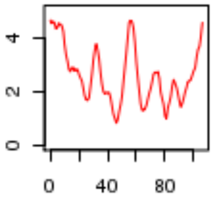

QMEAN4 global scores:

The QMEAN4 score is a composite score consisting of a linear combination of 4 statistical potential terms (estimated model reliability between 0-1). The pseudo-energies of the contributing terms are given below together with their Z-scores with respect to scores obtained for high-resolution experimental structures of similar size solved by X-ray crystallography:

Scoring function term	Raw score	Z-score
C_beta interaction energy	-55.30	-0.08
All-atom pairwise energy	-2279.85	-0.65
Solvation energy	-15.68	1.04
Torsion angle energy	1.36	-3.38
QMEAN4 score	0.569	-2.26

If you publish results from QMEAN, please cite the following paper:
Benkert P, Biasini M, Schwede T. (2011). "Toward the estimation of the absolute quality of individual protein structure models." Bioinformatics, 27(3):343-50.

Local Model Quality Estimation:

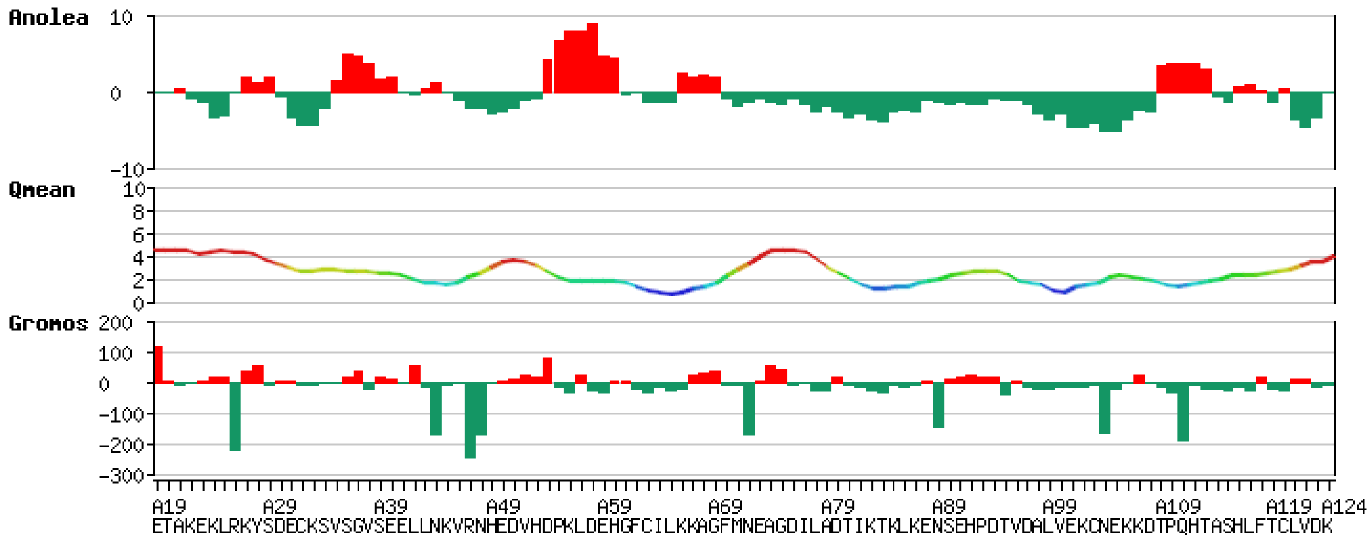

Alignment:

```
TARGET     1       ETAKEKLR KYSDECKSVS GVSEELLNKV RNHEDVHDPK LDEHGFCILK
1c3y_1     1       etpreklk qhsdackaes gvseeslnkv rnreevddpk lkehafcilk

TARGET                         hhhhhh        hhhhh            h hhhhhhhhhh
1c3y_1                          hhhhh        hhhhh            h hhhhhhhhhh


TARGET     49    KAGFMNEAGD ILADTIKTKL KENSEHPDTV DALVEKCNEK KDTPQHTASH
1c3y_1     49    ragfidasge fqldhiktkf kensehpekv ddlvakcavk kdtpqhssad

TARGET           hh            hhhhhhhh h         h hhhhhhh         hhhhhh
1c3y_1           hh            hhhhhhhh h         h hhhhhhh         hhhhhh


TARGET     99    LFTCLVDKK
1c3y_1     99    ffkcvhdnrs

TARGET
1c3y_1           h
```

Modeling Log:

```
3.70 (SP3)
Loading Template: 1c3y_1.pdb
Loading Raw Sequence
Loading Alignment: ./NXXX.align.submit.fasta
Removing HET groups from template structure
Refining Raw Sequence Alignment
ProModII: doing simple assignment of backbone
ProModII: adding blocking groups
Adding Missing Sidechains
AddPolar H
BuildDeletetedLoopsModel
Optimizing Sidechains
Adding Hydrogens
Optimizing loops and OXT (nb = 1)
```

```
Final Total Energy:       -1832.706 KJ/mol
Dumping Sequence Alignment
```

Template Selection Log:

```
 - Start SMR-Pipeline in automated mode on BC2-cluster at Thu May  3 12:46:29 2012


 - Start BLAST for highly similar template structure identification
 - Blast template search was successfull, number of templates selected for modeling:  1; entering modeling mo

 - Send 1 target-template alignments for modeling

 - @@@@@@@@@@@@@@@@@@@@@@@@@@@@@@@@@@@@@@@@@@@@@@@@@@@@@@@@@@@@@@@@@@@@@@@@@@@@@@@@
 -             ******************************************************************
 - building model based on 1c3yA (19-125) was successfull
 - Workspace Pipeline parameter

   Cut-off parameters to model the target based on a BLAST target-template alignment
    Evalue :                                     0.0001
    Minimum Template size (aa) for ranking :     25
    Minimum Sequence identity :                  60

   Cut-off parameters to model the target based on a HHSearch target-template alignment
    Evalue :                                     0.0001
    Probability :                                50
    MAC :                                        0.3

   Parameters for model selection
    Minimal number of uncovered target
     residues after BLAST to run HHSEARCH :      50
    Minimal number of uncovered target
     residues to model an additional template :  25

 - Finish SMR-Pipeline in automated mode on BC2-cluster at Thu May  3 12:46:38 2012
```

Quaternary Structure Annotation of the Template
1c3y is annotated as MONOMER
The template structure (1c3y) was solved by NMR and does not contain any quaternary structure annotation
The template is annotated by the number of chains found in the PDB file

Quaternary Structure Modelling of the Target Protein
The quaternary structure can be assumed to be identical
Model was succesfully built as MONOMER.

Ligand Modeling Log: Template's ligands section
Template without ligands.
The template contains ligands that are not yet part of the pipeline. Ligands which are currently assessed are listed in the help page.
No ligands were included in the model.

References: If you publish results using SWISS-MODEL, please cite the following papers:

- Arnold K., Bordoli L., Kopp J., and Schwede T. (2006). The SWISS-MODEL Workspace: A web-based environment for protein structure homology modeling. Bioinformatics, 22,195-201.
- Schwede T, Kopp J, Guex N, and Peitsch MC (2003) SWISS-MODEL: an automated protein homology-modeling server. Nucleic Acids Research 31: 3381-3385.
- Guex, N. and Peitsch, M. C. (1997) SWISS-MODEL and the Swiss-PdbViewer: An environment for comparative protein modeling. Electrophoresis 18: 2714-2723.

SWISS-MODEL is developed by the Protein Structure Bioinformatics group at the SIB - Swiss Institute of Bioinformatics & the Biozentrum University of Basel. © 2010.
